# Enhancing ECG classification with continuous wavelet transform and multi-branch transformer

**DOI:** 10.1016/j.heliyon.2024.e26147

**Published:** 2024-02-21

**Authors:** Chenyang Qiu, Hao Li, Chaoqun Qi, Bo Li

**Affiliations:** School of Information Technology, Yunnan University, Kunming, China

**Keywords:** Arrhythmia, Multi-branch transformer, Continuous wavelet transform, Convolutional neural network, Time-series feature map

## Abstract

**Background:**

Accurate classification of electrocardiogram (ECG) signals is crucial for automatic diagnosis of heart diseases. However, existing ECG classification methods often require complex preprocessing and denoising operations, and traditional convolutional neural network (CNN)-based methods struggle to capture complex relationships and high-level time-series features.

**Method:**

In this study, we propose an ECG classification method based on continuous wavelet transform and multi-branch transformer. The method utilizes continuous wavelet transform (CWT) to convert the ECG signal into time-series feature map, eliminating the need for complicated preprocessing. Additionally, the multi-branch transformer is introduced to enhance feature extraction during model training and improve classification performance by removing redundant information while preserving important features.

**Results:**

The proposed method was evaluated on the CPSC 2018 (6877 cases) and MIT-BIH (47 cases) ECG public datasets, achieving an accuracy of 98.53% and 99.38%, respectively, with F1 scores of 97.57% and 98.65%. These results outperformed most existing methods, demonstrating the excellent performance of the proposed method.

**Conclusion:**

The proposed method accurately classifies the ECG time-series feature map, which holds promise for the diagnosis of cardiac arrhythmias. The findings of this study are valuable for advancing the field of automatic ECG diagnosis.

## 1 Introduction

The electrocardiogram (ECG) is a common method of cardiac monitoring and plays a crucial role in the diagnosis and monitoring of arrhythmias [[1], [2], [3], [4]]. However, the complexity and variability of ECG signals make the classification task extremely challenging. Traditional ECG classification methods rely on manual feature extraction and classifier design, which are prone to human error and require extensive expertise and experience [5]. As a result, there is a pressing need for more advanced and automated methods of ECG classification that can improve diagnostic accuracy and reduce the workload of medical professionals.

Continuous wavelet transform (CWT) [6] is a signal analysis technique widely used to transform ECG signals into 2D images, which can decompose ECG signals into wavelet coefficients of different scales and orientations, and then efficiently extract useful feature information by combining these coefficients into time-series feature map. Transformer [7] is a powerful deep learning tool that can adaptively learn important feature information and improve the classification ability of the model.

In recent years, with the development of deep learning technology, ECG classification methods based on deep learning have gradually received wide attention. For instance, Acharya et al. [8] proposed a deep convolutional neural network (DCNN) [9] ECG classification method, which can automatically detect cardiovascular diseases such as myocardial infarction. However, the method cannot learn from past decisions and features, which can lead to clusters being mixed with other classes and affecting classification results. To address this issue, Hannun et al. [10] introduced a long short-term memory (LSTM) network [11] ECG classification method that can also automatically detect arrhythmias, but requires data preprocessing. Recently, the transformer has been widely used in the field of ECG classification. For example, Ramkumar et al. [12] developed a bidirectional long-term and short-term memory network that utilizes an attention mechanism to adaptively learn important ECG features, enabling the classification of various arrhythmias. However, this method requires complex data preprocessing operations and consumes significant computational resources and time, which can affect the model's accuracy. Yang et al. [13] used a multi-view approach to fuse different lead features and used a multi-scale convolutional neural network to obtain ECGs of different scales with temporal characteristics. Lai et al. [14]collected a large number of wearable 12-lead ECG datasets and achieved real-time intelligent diagnosis through four data enhancement operations and a self-supervised learning classification framework. In addition, Han et al. [15] used the Gramian Angular Field (GAF) to map the original ECG signal into feature map, introduced a multi-instance learning (MIL) method to avoid information loss, and used a feature fusion method based on the attention mechanism to achieve accurate classification.

Although effective, the ECG classification methods mentioned above have certain limitations. Firstly, 1DCNN or LSTM-based [16,17] methods require signal preprocessing and manual feature selection, which can be prone to human error and cannot learn past decisions and features. Secondly, 2DCNN or transformer-based [[18], [19], [20], [21], [22]] methods, while not requiring preprocessing operations, struggle to extract complex relationships between time steps in the time-series feature map and high-level time-series features. To overcome these challenges, this paper proposes an ECG classification method that utilizes CWT and multi-branch transformer. This method converts the original ECG signal into time-series feature map using CWT, eliminating the need for complicated preprocessing. Additionally, the multi-branch transformer is introduced to enhance feature extraction and improve classification performance by removing redundant information while preserving important features. Experimental results demonstrate that the proposed method achieves excellent performance in ECG classification tasks.

This work makes a twofold contribution. Firstly, a multi-branch transformer-based ECG classification method is introduced. The method utilizes convolution to extract deep features of the image, and multi-branch transformer to extract global features of the image. This operation enables the accurate classification of various arrhythmias. Secondly, an enhanced multi-headed self-attention mechanism is used to compute only the useful features in the time-series feature map, which improves the efficiency while still focusing on the subtle changes of the signal.

The rest of this paper is structured as follows. The proposed method is described in Section 2. Section 3 describes the dataset used in this study with data enhancement and partitioning. The results of the study are analyzed in Section 4, and Section 5 discusses the results. Finally, conclusions and an outlook for future research are given in Section 6.

## 2 Methodologies

### 2.1 Overall architecture of ECG classification method

**Fig. 1:**
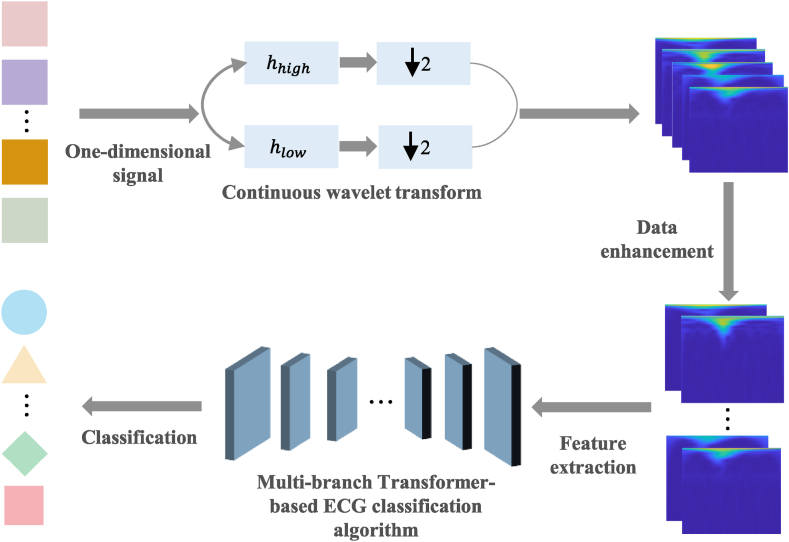
The illustration of the proposed classification method. The figure shows the flowchart of the proposed classification method, including data preprocessing, data Enhancement, feature extraction, and classification.

The overall flow of the proposed ECG classification method based on CWT and multi-branch transformer is shown in Fig. 1. Firstly, the obtained electrocardiogram signal is mapped into a time-series feature map through continuous wavelet transform. Then, the obtained image is data enhanced, and the Multi-branch Transformer algorithm is used for feature extraction and classification, ultimately obtaining different categories of arrhythmia.

### 2.2 Continuous wavelet transform

The wavelet transform can be divided into two forms: Continuous Wavelet Transform (CWT) [6] and Discrete Wavelet Transform (DWT) [23]. It is a multi-scale analysis technique that decomposes the original signal into approximate and detailed coefficients at varying scales. The approximate coefficients represent the low-frequency part of the signal, while the detail coefficients represent the high-frequency part of the signal. CWT is faster to compute than DWT, has better localization and multi-resolution characteristics, and is, therefore, used in this paper to convert ECG signals into time-series feature maps. The calculation of CWT is depicted in Eq. (1).

(1) $$C_{a,b}=\frac{1}{\sqrt{\left|a\right|}}\int_{-\infty }^{+\infty }x\left(t\right)\psi^{*}\left(\frac{t-b}{a}\right)dt$$

where x(t) is the input signal, ψ(t) is the wavelet basis function, a and b are the scale and translation parameters, and * denotes the complex conjugate. $C_{a,b}$ are the wavelet coefficients representing the components of the signal at scale a and position b. CWT has a better adaptive capability in terms of time-frequency resolution, which can better highlight the local feature of the actual ECG signal.

### 2.3 Multi-branch transformer-based ECG classification algorithm

**Fig. 2:**
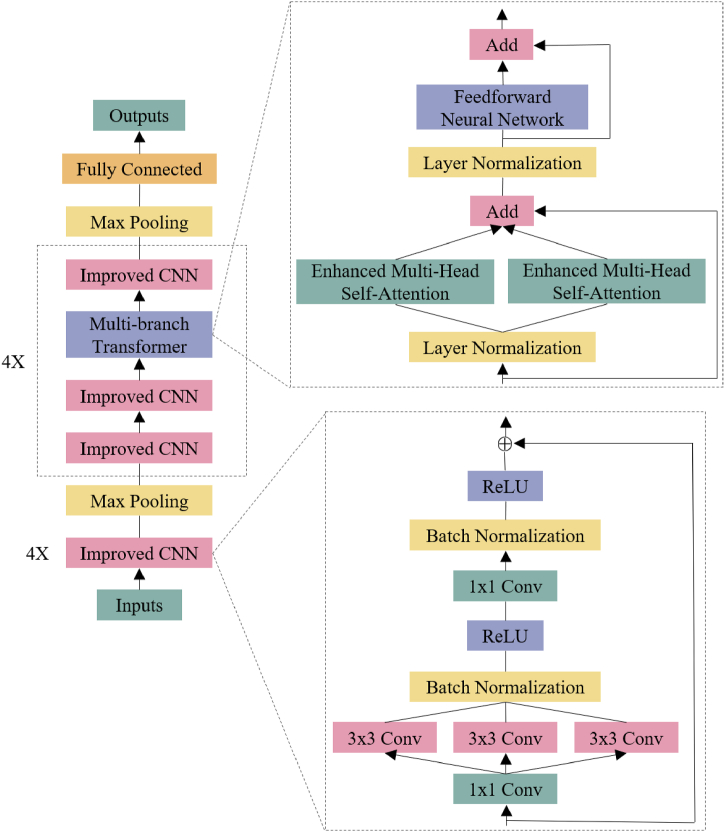
Overall architecture of ECG classification algorithm. The figure shows the overall architecture of the proposed ECG classification algorithm, which consists of two main parts: the improved CNN and the multi-branch transformer.

The overall architecture of the multi-branch transformer-based ECG classification algorithm is illustrated in Fig. 2. The algorithm takes the original ECG signal as input and converts it into time-series feature map using CWT. In the feature extraction module, local features are extracted using improved CNN, and the extracted features are then weighted and fused by multi-branch transformer to enhance the classification performance. Finally, a fully connected layer maps the features of each arrhythmia class to obtain the final classification results. This algorithm not only has the transformer's ability to extract global features from images but also avoids its dependence on large datasets.

#### 2.3.1 Improved CNN

The proposed algorithm utilizes improved CNN for the local feature extraction module, comprising four convolutional blocks. Each block contains multiple convolutional layers and batch normalization layers. The inputs and outputs of the convolutional blocks are connected through residuals, which boosts network performance without increasing the parameter count. Fig. 2 illustrates the network structure.

In practical ECG classification tasks, it is important to consider time efficiency. To reduce the computational and storage overhead of the model, the improved CNN uses depthwise separable convolution (DSC) [24] instead of the standard convolutional layer. This approach can significantly decrease the number of parameters and computation of the model, leading to improved classification performance. The calculation process is demonstrated in Eq. (2).

(2) F(x)=BN(ReLU(DSC(Conv(x))))

#### 2.3.2 Multi-branch transformer

To improve the robustness and classification performance of the network in extracting ECG features, this paper adopts a cross structure comprising convolution and transformer blocks in the global feature extraction module. The multi-branch transformer structure can be seen in Fig. 2.

The multi-branch transformer layer enhances the input features for computation, while also establishing connections between different categories. It achieves this by connecting and interacting with each category to obtain ${EMHSA}_{1}\left(X\right)$. The final result is obtained by passing the original sequence through the second multi-headed attention module, and then ${\;EMHSA}_{1}\left(X\right)$ acts as the residual connection to get $X_{0}$. For specific details on the calculation process, please refer to Eq. (3).

(3) $$X_{0}={EMHSA}_{1}\left(X\right)+{EMHSA}_{2}\left(X\right)+X$$

In the equation above, the outputs of the two branches are connected with residuals, which allows the modeling of the inputs at different levels and granularities. Finally, the feedforward neural network (FNN) [25] is added to obtain the final output of this transformer block. As shown in Eq. (4).

(4) $$Y=\mathrm{LN}\left(\;X_{0}\right)+FNN\left(\;\mathrm{LN}\left(X_{0}\right)\right)$$

The multi-branch transformer can capture various relationships in different branches and flexibly control the flow of information and interactions between them. This ability allows for a more comprehensive description of the interactions between sequences, improving the learning and generalization capabilities of the model.

#### 2.3.3 Enhanced multi-headed self-attention mechanism

The traditional transformer employs a multi-headed self-attention mechanism [7] that captures only intra-sequence dependencies. It fails to consider inter-sequence or long-range dependencies across time steps and often requires considerable computational resources. Additionally, when dealing with time-series feature maps, it is inefficient and redundant to compute attention between each pixel in pairs. This is because the local regions surrounding each pixel share similar features.

**Fig. 3:**
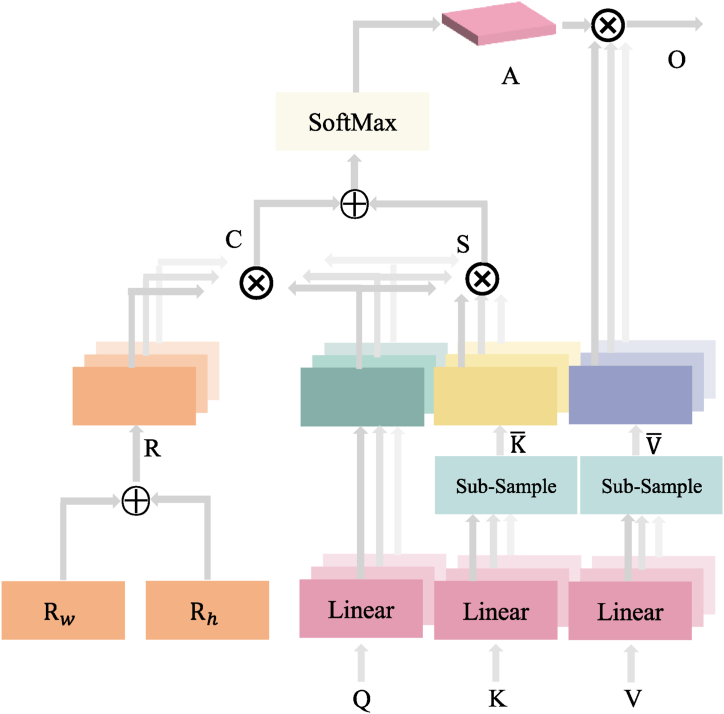
E-MHSA structure diagram. The figure shows the structure diagram of the proposed E-MHSA (Enhanced Multi-Head Self-Attention). The model consists of several layers of self-attention modules, where each module has multiple attention heads. The input to the model is a sequence of embeddings, which are transformed by the self-attention modules to capture the interactions between different parts of the sequence. In addition, the model also incorporates relative position coding to improve its performance. The proposed E-MHSA model has been shown to achieve state-of-the-art results on ECG classification tasks.

To address the issues mentioned above, this paper proposes the integration of enhanced multi-headed self-attention (E-MHSA) into ECG classification networks. Unlike the traditional multi-headed self-attention mechanism, E-MHSA not only captures intra-sequence dependencies but also has the potential to handle inter-sequence and long-range dependencies in the input feature graph. Moreover, it exhibits high computational efficiency and requires minimal memory consumption. The architecture of E-MHSA is illustrated in Fig. 3.

Suppose the input sequence is $X\in R^{n\times d},R_{w}\in R^{w\times d},R_{h}\in R^{h\times d}$, where n is the sequence length, and d is the feature dimension. A linear transformation of the input sequence yields $Q=XW_{Q},\bar{K}=XW_{K},\bar{V}=XW_{V}$, where $W_{Q},W_{K},W_{V}\in R^{d\times h}$ is the weight matrix of the linear transformation, and h is the number of heads. Then, the weights of $Q,\bar{K},\bar{V}$, respectively by the number of heads h are sliced, and the dimension of each head is d/h. First, calculate the attention score matrix $S\in R^{h\times n\times n},C\in R^{h\times n\times n}$, see Eq. (5).

(5) $$\left\{ \begin{array}{c} S_{i,j}=\frac{Q_{i}{\bar{K}}_{j}^{T}}{\sqrt{d/h}}\\ C_{i,j}=\frac{\left(R_{w}+R_{h}\right)Q_{i}^{T}}{\sqrt{d/h}} \end{array}\right.$$

Then, the attention score matrix is subjected to softmax operation to obtain the attention matrix $A\in R^{h\times n\times n}$, see Equation (6).

(6) $$A_{i,j}=\frac{e^{S_{i,j}}}{\sum_{k=1}^{n}e^{S_{i,k}}}+\frac{e^{C_{i,j}}}{\sum_{k=1}^{n}e^{C_{i,k}}}$$

Finally, the attention matrix A with the sliced $\bar{V}$ matrix is weighted and summed to obtain the output feature matrix $O\in R^{n\times d}$, see Eq. (7).

(7) $$O=concat\left({head}_{1},{head}_{2},\cdots ,{head}_{h}\right)W_{O}$$

In the above equation, the ${head}_{i}=\sum_{j=1}^{n}A_{i,j}{\bar{V}}_{i,j}$ represents the output of the first i output of the first attention head, and concat denotes the merging of the output from each attention head in the feature dimension, while $W_{O}\in R^{d\times d}$ is the weight matrix of the output matrix.

E-MHSA exhibits improved performance and computational efficiency in handling long sequences and high-dimensional data by reducing the number of attention heads while ensuring accuracy. This allows for efficient processing of large amounts of data without sacrificing performance.

#### 2.3.4 Relative position coding

To classify ECG time-series features, this paper utilizes relative position coding. This method offers superior accuracy in coding positional relationships, as well as greater robustness to time-series shifts. Additionally, it enhances generalization to new datasets and tasks while effectively modeling non-linear relationships. These benefits contribute to improved accuracy and robustness in classification results, particularly when coupled with E-MHSA. As illustrated in Fig. 3, R represents the relative position coding.

## 3 Experiment setup

### 3.1 Experimental dataset

In this study, we first convert the original ECG signal into time-series feature map using CWT, followed by feature extraction and classification to obtain the final results. The experiments were conducted using the China Physiological Signals Challenge 2018 (CPSC 2018) [26] and ECG recordings from the MIT-BIH [27], the latter were developed in collaboration with MIT and Beth Israel Medical Center. These datasets were used to train, test, and validate the robustness of our ECG classification algorithm. The arrhythmia categories in both datasets were independently annotated by two or more cardiac experts to ensure their authority. Table 1 shows the number of cases and class distribution for each dataset.

**Table 1:** Assigning patients to categories. The letter G stands for group and the first row G1-G9 corresponds to the 9 categories in CPSC 2018 (Male 3699, Female 3178). The second row G1-G5 corresponds to the 5 categories in MIT-BIH (Male 25, Female 22).

Dataset	G1	G2	G3	G4	G5	G6	G7	G8	G9	Total
CPSC 2018	918	1098	704	207	1695	574	653	826	202	6877
MIT-BIH	27	5	5	5	5	–	–	–	–	47
#patients	945	1103	709	212	1700	574	653	826	202	6924
#instances	2445	2603	2209	1712	3200	574	653	826	202	14,424

**Fig. 4:**
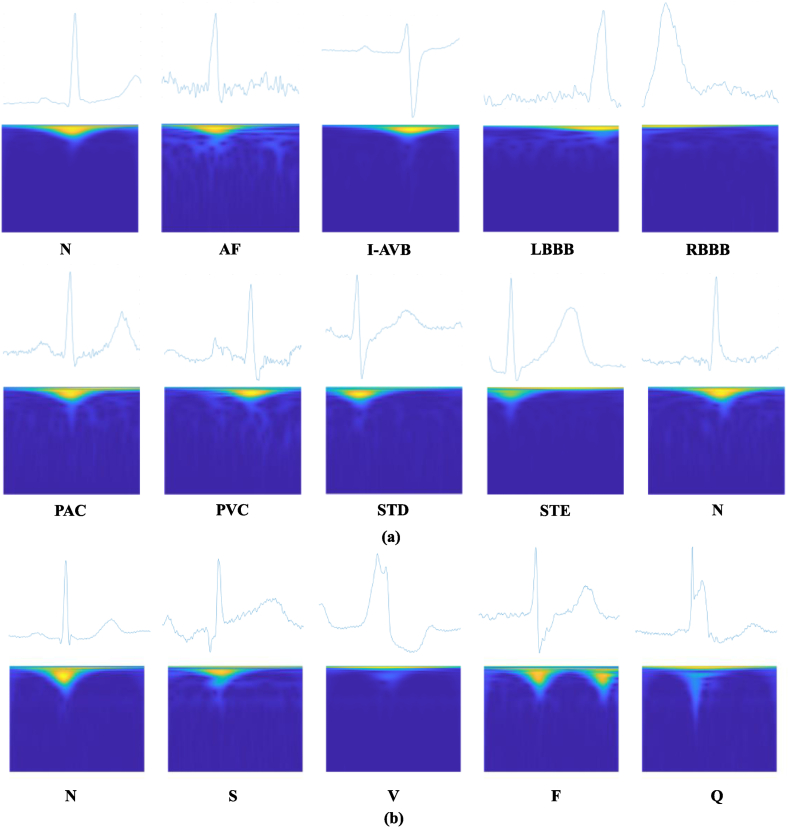
Time-series feature map after CWT. (a) For CPSC 2018 dataset and (b) for MIT-BIH dataset. The figure shows the time-series feature maps obtained using continuous wavelet transform (CWT) for two different ECG datasets: CPSC 2018 and MIT-BIH. CWT is applied to the raw ECG signals to obtain a time-frequency representation, which captures both the temporal and spectral information of the signals. The resulting feature maps are then used as inputs to the proposed algorithm for ECG classification. The figure demonstrates the differences in the feature maps between the two datasets, highlighting the need for dataset-specific feature engineering in ECG analysis.

The CPSC 2018 dataset [Fig. 4 (a)] consists of 6877 12-lead ECG recordings, with durations ranging from 6 to 60 s and a sampling rate of 500 Hz. This dataset includes nine categories: Normal (N), Atrial fibrillation (AF), First-degree atrioventricular block (I-AVB), Left bundle branch block (LBBB), Right bundle branch block (RBBB), Premature atrial contraction (PAC), Premature ventricular contraction (PVC), ST-segment depression (STD), and ST-segment elevated (STE).

Furthermore, the MIT-BIH dataset [Fig. 4 (b)] includes 48 half-hour ECG recordings, including 23 arrhythmias and normal sinus rhythm, with a sampling rate of 360 Hz and two signal channels per recording. According to the ANSI/AAMI EC57 classification proposed by the Association for the Advancement of Medical Instrumentation (AAMI), the MIT-BIH arrhythmia database is divided into five categories: Normal (N), Supraventricular premature beats (S), Ventricular premature beats (V), Atrial fibrillation (F), and Unknown category (Q).

Fig. 4 displays the time-series feature maps obtained after the CWT transformation. Generally, information that is not easily obtained in the time domain can be obtained in the frequency domain. Although some 1D signal to 2D image methods can achieve this effect, CWT can more accurately capture the frequency domain information of the timing signal without losing the time domain information. Moreover, in ECG signals, some waveforms appear random in 1D signals but have more distinct features or show strong regionality in 2D images. Therefore, converting the original signal into time-series feature map using CWT is more conducive to feature extraction.

### 3.2 Dataset segmentation and data enhancement

**Fig. 5:**
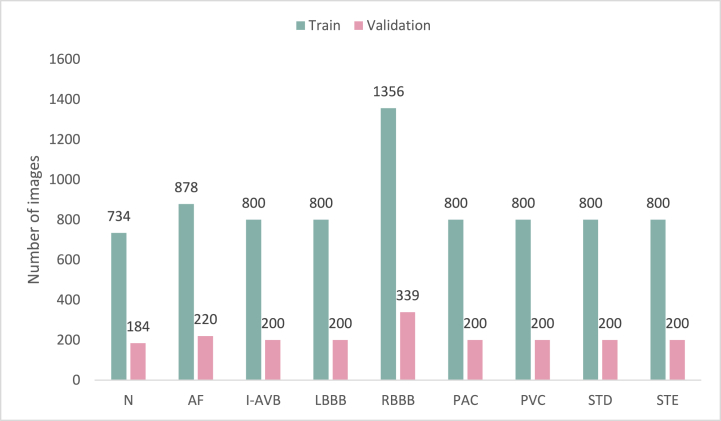
Distribution of images across the 9 different classes. This figure depicts the distribution of images across the nine different classes in the CPSC 2018 dataset, which was utilized in the ECG classification task after data enhancement. The y-axis indicates the number of images, while the x-axis represents the nine different classes. The largest class is the RBBB class, consisting of 1695 images, whereas the smallest class is the N class, which comprises only 908 images. It is worth noting that class imbalance is a common issue in image classification tasks that can have a negative impact on the performance of the network. Therefore, to overcome this problem, appropriate data enhancement techniques and class weighting strategies are often employed.

The dataset used in this study is not evenly distributed, which is mainly due to patient privacy and the complex labeling task. To ensure that the neural network is not overfitting and to improve the overall performance of the classification algorithm, data enhancement is necessary to balance the number of normal and arrhythmia analogies. This paper employs various data augmentation techniques during the data preprocessing stage, such as brightness adjustment, rotation, horizontal flipping, scaling, and cropping, to increase the diversity and richness of the data, and enhance the adaptability of the model to different datasets. Moreover, the training set is approximately 8 times larger than the validation set, as illustrated in Fig. 5.

### 3.3 Study environment and parameter settings

The study discussed in this paper utilized PyCharm as the integrated development environment and the deep learning framework Pytorch. A detailed summary of the hardware configuration and software environment used in the study is presented in Table 2. The input image size was set to 224 × 224, with a fixed learning rate of 0.0001 used during training. The training process was conducted for 50 rounds (Epoch), with each training batch size set at 32, and the optimization algorithm used was adaptive gradient descent (Adam).

**Table 2:** Experimental environment.

Hardware or Software	Version or Model
Operating System	Windows 11
CPU	Intel Core i7 12,700
GPU	NVIDIA GeForce RTX 3070Ti
Graphics Memory	8G
Operating Memory	16G
Integrated Development Environment	PyCharm 2022.1
Programming Languages	Python 3.9
Deep Learning Framework	Pytorch 1.11
CUDA	CUDA 11.4

### 3.4 Evaluation indicators

In the experiment, the algorithm is evaluated using Accuracy (Acc), Precision (Pre), Recall (Rec), and F1-score as evaluation indicators. The formulas for calculating these metrics are provided in Eqs. (8), (9), (10), (11).

(8) $$Acc=\frac{TP+TN}{TP+FN+FP+TN}$$

(9) $$Pre=\frac{TP}{TP+FP}$$

(10) $$Rec=\frac{TP}{TP+FN}$$

(11) $$F1=\frac{2\times Pre\times Rec}{Pre+Rec}$$

where TP, TN, FP, and FN represent the number of true positives, true negatives, false positives, and false negatives predicted by the model for positive and negative classes. Moreover, a higher F1 score indicates better classifier performance and a score closer to 1 is considered optimal.

## 4 Results

### 4.1 Comparison study of different wavelet transform

In this section, we first used DWT and CWT to map the original electrocardiogram signals into time-series feature maps and tested the performance of the classifier on the CPSC 2018 dataset. From Table 3, it can be seen that CWT can transform signals at any time scale, providing richer frequency information, and is suitable for ECG signal processing and multi-scale analysis.

**Table 3:** The comparison of Discrete Wavelet Transform (DWT) and Continuous Wavelet Transform (CWT).

Indicators (%)	DWT	CWT
Acc	98.42	98.53
Pre	98.01	98.19
Rec	96.58	96.95
F1	97.22	97.57

### 4.2 Comparison study of different models

**Fig. 6:**
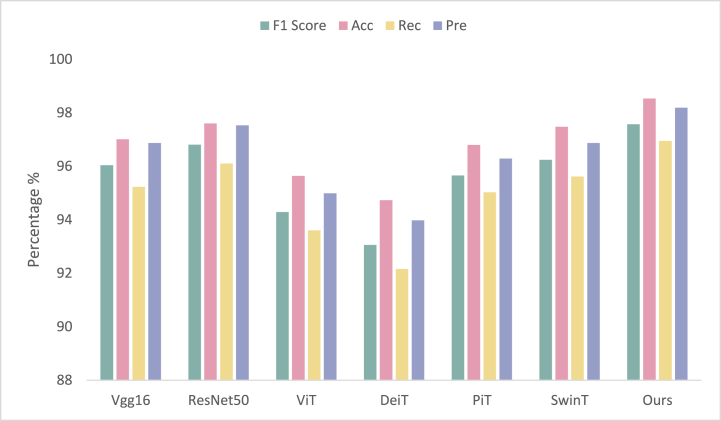
Comparison studies of different models. The figure shows a comparison of the performance of different ECG classification tasks. The y-axis represents the evaluation metric used to measure the performance, and the x-axis represents the different algorithms being compared. This figure shows the advantages of our algorithm.

In this experimental section, we validate the proposed ECG classification algorithm on the CPSC 2018 arrhythmia public dataset. To demonstrate the superiority of our approach, we compare it not only with Vgg16 [28] and ResNet50 [29], which use a convolutional neural network as the infrastructure but also with several vision transformer-based classification algorithms, namely Vision transformer (ViT) [30], Data-efficient image transformer (DeiT) [31], Pooling-based vision transformer (PiT) [32] and Swin transformer (SwinT) [33]. It is worth noting that all models were trained without the use of pre-trained weights. Fig. 6 presents the comparison results of the different algorithms.

**Fig. 7:**
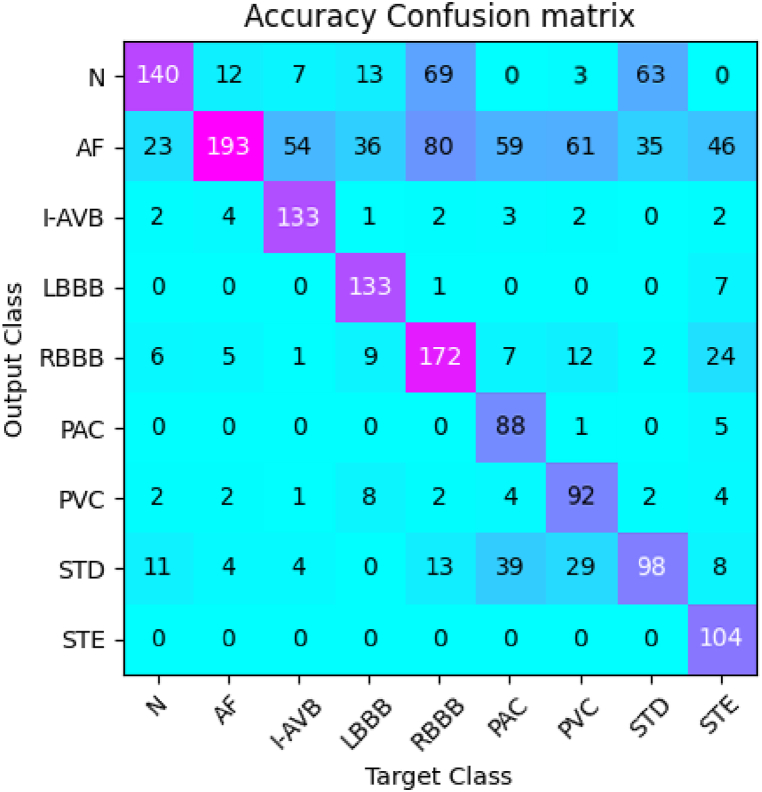
Confusion matrix of the CPSC 2018 dataset. This figure shows the performance of the proposed method on the CPSC 2018 dataset.

Based on Fig. 6, Fig. 7, it is evident that the proposed algorithm delivers superior performance across all evaluation metrics, particularly in accuracy, with an impressive score of 98.53%, which is approximately 2.5% higher than the other models on average. Notably, the Vgg16 and ResNet50 models performed relatively well, with accuracies of 97.01% and 97.60%, respectively. On the other hand, the transformer family models exhibited average performance, with DeiT and ViT recording accuracy scores of 94.73% and 95.64%, respectively. Furthermore, our proposed algorithm outperformed PiT and SwinT, which achieved accuracy rates of 96.80% and 97.48%, respectively. In terms of precision, recall, and F1 score, our algorithm delivered superior results of 98.19%, 96.95%, and 97.57%, respectively, compared to other models. These findings highlight the remarkable performance advantage of our proposed algorithm for ECG classification problems, providing accurate and reliable diagnostic support for heart diseases in the field of clinical diagnosis and monitoring.

### 4.3 Ablation study

To validate the efficiency of the algorithm proposed, a range of ablation experiments have been carried out in this section, utilizing the enhanced CNN, multi-branch transformer (MT), and E-MHSA. These experiments have all been conducted on the CPSC 2018 dataset.

**Fig. 8:**
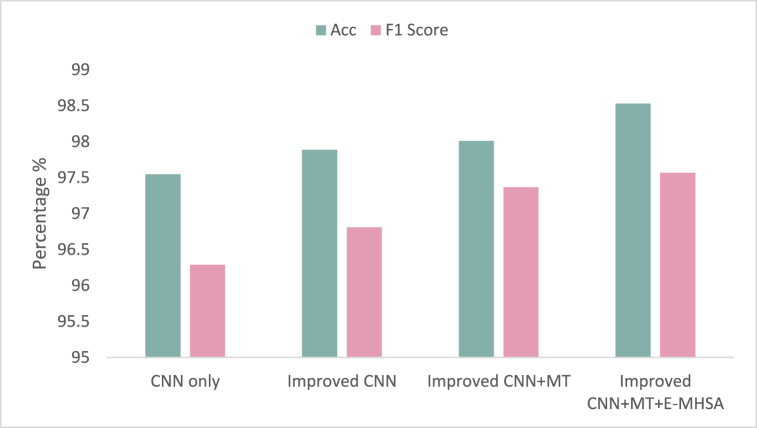
Ablation studies of various parts. The figure shows the results of ablation studies, which involve systematically removing or disabling various parts of the proposed algorithm to evaluate their impact on performance. The y-axis represents the evaluation metric used to measure the performance. The x-axis represents the different ablation conditions being compared. The figure demonstrates the relative contributions of different parts of the algorithm, allowing researchers to identify the most important components and optimize their design.

In Fig. 8, we compare the classification performance of different parts, with the vertical axis representing the classification accuracy. The results demonstrate that using the improved CNN leads to a 0.34% increase in classification performance. When incorporating the multi-branch transformer, there is a further improvement of 0.12%. Moreover, by replacing the original MHSA with E-MHSA in the multi-branch transformer, the accuracy reaches the highest point of 98.53%. These findings suggest that the proposed algorithm is effective in extracting crucial point information from the time-series feature map, leading to a superior classification performance.

### 4.4 Robustness study

**Fig. 9:**
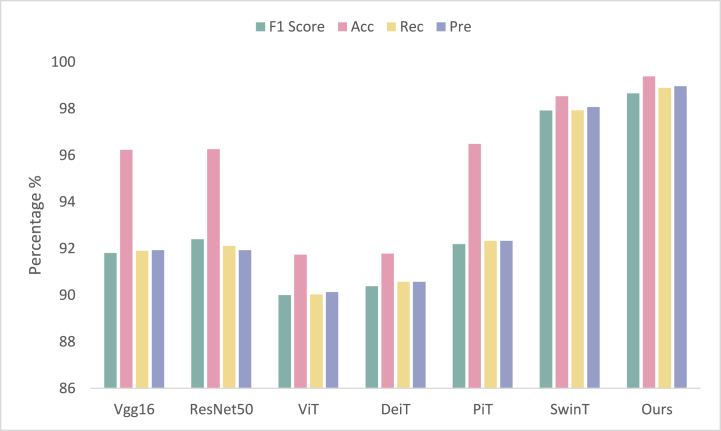
Robustness studies of the proposed algorithm. The figure shows the results of robustness studies, which involve testing the performance of our proposed algorithm on the MIT-BIH ECG benchmark dataset. The y-axis represents the evaluation metric used to measure the performance, the x-axis represents the different algorithms being compared. The figure demonstrates the ability of the proposed algorithm to maintain high performance, indicating its robustness and generalizability.

**Fig. 10:**
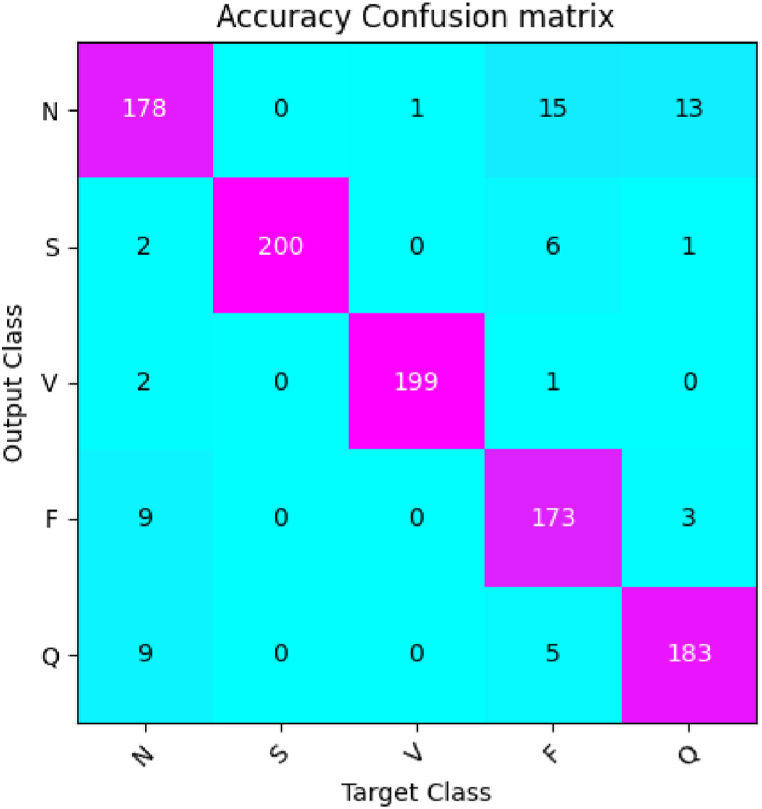
Confusion matrix of the MIT-BIH dataset. This figure shows the performance of the proposed method on the MIT-BIH dataset.

The study employed the same classification algorithm, parameter settings, and evaluation metrics as the previous paper. It is worth noting that all models were trained without using pre-trained weights. The comparison results of different algorithms are presented in Fig. 9. As depicted in Fig. 9, Fig. 10, the proposed algorithm outperformed other algorithms on the MIT-BIH public ECG dataset with an accuracy rate of 99.38%, which is considerably higher than that of other algorithms. Furthermore, it exhibited the best results concerning precision, recall, and F1 score. Consequently, the proposed algorithm demonstrated excellent performance and robustness on various datasets and effectively improved the accuracy and efficiency of ECG signal classification.

### 4.5 Comparison of different models with and without noises

We conducted a thorough analysis of the model's ability to handle noise in ECG signals. To address the need for extended sequence data, we selected the MIT-BIH dataset, where each record spans 1800 s. Table 4 illustrates that, in the presence of noise in the original signal, both convolution and Transformer-based algorithms exhibit a declining performance trend. Nevertheless, our algorithm sustains an accuracy of 98.19% in such situations. Subsequently, following the denoising of the original signal, our algorithm exhibits a substantial performance enhancement, reaching an accuracy of 99.38%, representing a 1.19% increase compared to the pre-denoising stage. Consequently, our algorithm showcases exceptional performance in managing extended sequences and noisy ECG signals.

**Table 4:** Comparison of different models with and without noises.

Methods	Noises				Without Noises			
	F1 (%)	Acc (%)	Rec (%)	Pre (%)	F1 (%)	Acc (%)	Rec (%)	Pre (%)
Vgg16 [28]	91.26	96.13	91.19	91.33	91.81	96.23	91.90	91.93
ResNet50 [29]	91.31	96.43	91.83	91.46	92.39	96.26	92.10	91.93
ViT [30]	89.07	90.87	89.17	89.17	90	91.73	90.03	90.13
DeiT [31]	89.56	90.53	89.70	89.70	90.38	91.78	90.57	90.57
PiT [32]	91.28	96.59	91.33	91.33	92.19	96.48	92.33	92.33
SwinT [33]	97.09	98.14	96.49	97.58	97.92	98.53	97.93	98.07
Proposed method	97.85	98.19	97.95	98.09	98.65	99.38	98.89	98.96

## 5 Discussion

In this research, we utilized the CWT method to transform the original one-dimensional ECG signal into time-series feature map. Additionally, Table 5 lists other relevant studies [[34], [35], [36], [37], [38], [39]]. Although some of these methods were not specifically intended for ECG signals, they all address the classification of time-series signals and thus have some reference value. The CWT method used in this paper achieved the highest classification accuracy of 99.38%, which is superior to other methods. By using CWT to convert ECG signals into time-series feature maps, the signal features can be more comprehensively described, leading to improved classification accuracy, generalization performance, and classifier robustness.

**Table 5:** Nine methods review. The methods include: Short Time Fourier Transform (STFT), Markov Transition Field (MTF), Gramian Angular Field (GAF), Recurrence Plots (RP), Motif Difference Field (MDF), Relative Position Matrix (RPM), Superlet Transform (SLT), Finite Difference Method (FDM). The Classifiers include: Denoised Diffusion Probabilistic Model (DDPM), Normal Cloud Representation CNN (NCR CNN).

Literature	Methods	Classifier	Datasets	Accuracy (%)
Kim et al. [34]	STFT	CNN	CinC 2017	99.26
Adib et al. [35]	MTF	DDPM	MIT-BIH	98.00
Ahmad et al. [36]	GAF	CNN	MIT-BIH	98.40
Mathunjwa et al. [37]	RP	DCNN	MIT-BIH	98.36
Zhang et al. [38]	MDF	CNN	TwoLeadECG	98.96
Hssayni et al. [39]	RPM	NCR CNN	TwoLeadECG	97.42
Tripathi et al. [40]	SLT	CNN	MIT-BIH	96.20
Kauppinen et al. [41]	FDM	CNN	MIT-BIH	98.01
Proposed method	CWT	CNN + MT	MIT-BIH	99.38

**Fig. 11:**
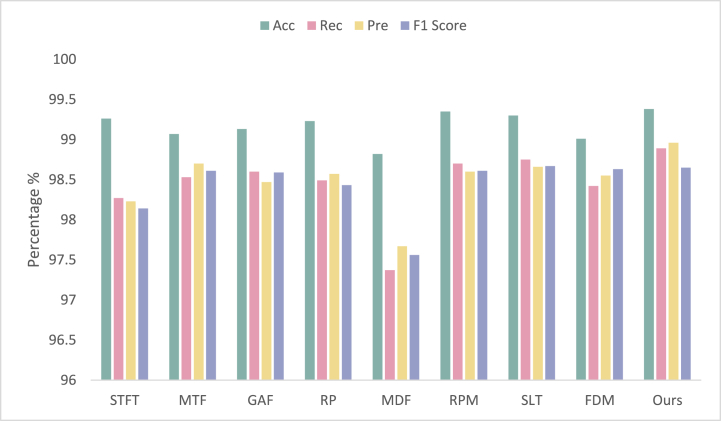
Comparison of the nine methods. The figure shows, on the MIT-BIH, the performance of our algorithm after converting 1D ECG signals to time-series feature maps using nine different methods. The y-axis represents the evaluation metrics used to measure the performance and the x-axis represents the different methods being compared. The figure shows the advantages of the CWT method.

To efficiently learn features in temporal spectrum images, this paper proposes a deep learning algorithm based on a multi-branch transformer for classifying arrhythmias. Using the nine methods outlined in Table 5, the algorithm extracts time-series feature maps, which are then used for feature extraction and classification. Our algorithm achieves a remarkable classification accuracy of up to 99.38%, as well as the highest F1 score, recall, and accuracy of 98.65%, 98.89%, and 98.96%, respectively, as demonstrated in Fig. 11. These results demonstrate the feasibility of our research solution and the effectiveness of the multi-branch transformer in extracting useful features and accurately classifying arrhythmias.

In the initial studies on classifying ECG signals, most of the methods used were based on one-dimensional signals [8,12,17], and a combination of multiple features, including temporal and frequency features, were empirically extracted. These methods required filtering the original signal, which resulted in the loss of significant information and some limitations. However, with the development of deep learning techniques, it has become possible to learn useful features from the original ECG signal, leading to the emergence of automatic arrhythmia classification based on deep learning, which is currently a popular research area [17]. One-dimensional convolutional neural networks have demonstrated good results in arrhythmia classification, but these methods only analyze the morphological features of ECG recordings, neglecting frequency and energy distribution information. To address these limitations, several studies have used the fusion of one-dimensional signals and time-series feature maps [[42], [43], [44]], allowing for encoding of key points of the original signal, but still requiring complex preprocessing and feature loss. In contrast, our study converts the original 1D signal directly into time-series feature map and employs an end-to-end approach for direct feature extraction of the time-series feature map, thereby improving classification accuracy while preserving the original features to a great extent. This approach differs from previous studies presented in Table 6.

**Table 6:** Literature review.

Literature	Features Set	Classifier	Results (%)	
			Rec	Acc
Acharya et al. [8]	R-peaks detection and11-layer deep neural network	Convolutional neural network	95.49	95.22
Ramkumar et al. [12]	Dual tree complex wavelet transform	Auto- Encoder and Bidirectional long short-term memory	99.43	97.15
Yang et al. [13]	Multi-view approach	Multi-scale convolutional neural network	95.47	–
Lai et al. [14]	–	Deep neural network	87.30	96.90
Han et al. [15]	Gramian angular field and multi-instance learning	Convolutional neural network and attention mechanism	–	–
Liang et al. [17]	–	Convolutional neural network- recurrent neural network	96.20	95.15
Vijayakumar et al. [42]	Denoising filter	Decision tree	94.35	96.50
Mazaheri et al. [43]	Denoising filter	Feed forward neural network	98.87	98.75
Le et al. [45]	–	Fusion transformer encoder	–	98.29
Che et al. [46]	Difference method and wavelet transform	Convolutional neural network and transformer	–	87.80
Wang et al. [47]	–	Convolutional neural network and transformer	97.40	–
Meng et al. [48]	Baseline drift	Lightweight fussing transformer	94.47	99.32
R Singh et al. [49]	Maximal overlap discrete wavelet transform	Bi-directional Long Short-Term Memory	–	95.40
Proposed method	Continuous wavelet transform	Multi-branch transformer and enhanced multi-headed self-attention	98.89	99.38

In recent studies on ECG signal classification tasks using transformer (Table 6), a combined approach of CNN and transformer has become the mainstream method for automatic ECG classification [[45], [46], [47], [50],^49^,^51^]. These approaches utilize the self-attention mechanism of the transformer to extract spatial information from images and convert them into sequential form for processing, thereby eliminating the need for manual feature extraction. However, these methods only employ a single transformer in their network structure, which limits the ability to fully exploit the correlation between time-series ECG signals and ignores important features such as nonlinearity and temporality.

The novel deep neural network proposed in Ref. [13], based on multi-view learning, integrates multi-scale convolutional blocks and coordinate attention modules to acquire high-quality electrocardiogram (ECG) features. It demonstrates excellent performance when dealing with long-term ECG signal records. In Ref. [14], self-collected ECG signal records were utilized for self-supervised learning to extract information from massive ECG data. Leveraging deep neural networks, this approach maintains high sensitivity and specificity in clinical testing. Reference [15] employed Graph Attention Filtering (GAF) to transform the raw ECG signals into images containing spatial-domain-related information between heartbeats, which were then combined with the original signals. Furthermore, a multi-instance learning (MIL) method was introduced to address data imbalance issues in long-term ECG signals. By utilizing multi-modal inputs, this model better allocates weights between instances and focuses more on information-rich instances.

Some studies have attempted to address this limitation by using a multi-branch transformer to implement the ECG classification task [44,48]. However, they have utilized the multi-headed self-attention mechanism (MHSA) of the original transformer for adaptive weighting of the entire image, which leads to poor computational efficiency. Since ECG signals are highly structured data in time-series feature maps, only a small fraction of the upper pixels contain useful information (Fig. 4). Pairwise attention computation between all pixels is therefore highly inefficient and redundant. In contrast, our study proposes a multi-branch transformer network with an enhanced multi-headed self-attention mechanism (Fig. 2, Fig. 3) that extracts only the relevant features from the image and focuses on subtle changes in the signal. This approach reduces computation and improves classification accuracy (Table 6), making it more suitable for realistic application scenarios. Compared to the methods mentioned above, our approach is more advantageous and efficient.

Although this study has yielded positive outcomes, it is essential to acknowledge its limitations. One of the primary constraints is the limited size of the experimental dataset, which may not be fully representative of the intricate nature of real-world scenarios. Furthermore, due to the concern for patient privacy, the scope of data collection had to be restricted, which has potentially resulted in a narrower focus of the study. Therefore, to validate the performance of the proposed algorithm, it is imperative to conduct further testing on larger and more diverse datasets. This will not only enhance the reliability of the study but also provide a more comprehensive understanding of the algorithm's efficacy in different contexts.

## 6 Conclusion

This study found that out of the nine commonly used methods for converting ECG signals into time-series feature maps, CWT exhibits the most robust encoding ability for signals, thereby facilitating feature extraction for classification algorithms. Moreover, the implementation of a multi-branch transformer and enhanced multi-headed self-attention mechanism leads to improved algorithm performance and generalization capabilities. Generally, this study has successfully demonstrated the automatic classification of cardiac arrhythmias, which could assist in the diagnosis and treatment of cardiovascular diseases while reducing physicians' workload.

In future work, we will further explore how to better handle the noise in the ECG signal while retaining more ECG signal characteristics. And use the data in the ECG cloud platform to continue training the model so that it can learn more arrhythmia features and improve the generalization performance of the model in the face of new long-term data.

## Funding statement

Hao Li was supported by the 10.13039/501100018531Yunnan Province Major Science and Technology Projects [202202AE090019].

## Data availability statement

Data will be made available on request.

## Additional information

No additional information is available for this paper.

## CRediT authorship contribution statement

**Chenyang Qiu:** Writing – review & editing, Writing – original draft, Visualization, Validation, Software, Resources, Project administration, Data curation. **Hao Li:** Supervision, Resources, Project administration. **Chaoqun Qi:** Software, Resources, Data curation. **Bo Li:** Data curation.

## Declaration of competing interest

The authors declare that they have no known competing financial interests or personal relationships that could have appeared to influence the work reported in this paper.
